# Minor surgery in general practice and effects on referrals to hospital care: Observational study

**DOI:** 10.1186/1472-6963-11-2

**Published:** 2011-01-04

**Authors:** Christel E van Dijk, Robert A Verheij, Peter Spreeuwenberg, Peter P Groenewegen, Dinny H de Bakker

**Affiliations:** 1 NIVEL, Netherlands Institute for Health Services Research, Utrecht, The Netherlands; 2 Utrecht University, Department of Sociology, Department of Human Geography, Utrecht, the Netherlands; 3 Tilburg University, Scientific Centre for Transformation in Care and Welfare (TRANZO), Tilburg University, Tilburg, The Netherlands

## Abstract

**Background:**

Strengthening primary care is the focus of many countries, as national healthcare systems with a strong primary care sector tend to have lower healthcare costs. However, it is unknown to what extent general practitioners (GPs) that perform more services generate fewer hospital referrals. The objective of this study was to examine the association between the number of surgical interventions and hospital referrals.

**Methods:**

Data were derived from electronic medical records of 48 practices that participated in the Netherlands Information Network of General Practice (LINH) in 2006-2007. For each care-episode of benign neoplasm skin/nevus, sebaceous cyst or laceration/cut it was determined whether the patient was referred to a medical specialist and/or minor surgery was performed. Multilevel multinomial regression analyses were used to determine the relation between minor surgery and hospital referrals on the level of the GP-practice.

**Results:**

Referral rates differed between diagnoses, with 1.0% of referrals for a laceration/cut, 8.2% for a sebaceous cyst and 10.2% for benign neoplasm skin/nevus. The GP practices performed minor surgery for a laceration/cut in 8.9% (SD:14.6) of the care-episodes, for a benign neoplasm skin/nevus in 27.4% (SD:14.4) of cases and for a sebaceous cyst in 26.4% (SD:13.8). GP practices that performed more minor surgery interventions had a lower referral rate for patients with a laceration/cut (-0.38; 95%CI:-0.60- -0.11) and those with a sebaceous cyst (-0.42; 95%CI:-0.63- -0.16), but not for people with benign neoplasm skin/nevus (-0.26; 95%CI:-0.51-0.03). However, the absolute difference in referral rate appeared to be relevant only for sebaceous cysts.

**Conclusions:**

The effects of minor surgery vary between diagnoses. Minor surgery in general practice appears to be a substitute for specialist medical care only in relation to sebaceous cysts. Measures to stimulate minor surgery for sebaceous cysts may induce substitution.

## Background

International comparative research shows that healthcare systems with a strong primary care orientation tend to have lower healthcare costs[1]. In the last years, strengthening of primary care is the focus of several countries[2]. In a recent report of the World Health Organisation (WHO) the importance of primary healthcare was emphasized [3]. Examples of countries with a strong primary care system are the UK, the Netherlands and Scandinavian countries. In these countries, GPs function as a gatekeeper to other healthcare providers and they decide on whether or not to refer patients for hospital treatment. Research also shows that within these countries, there is a great variation in GP referral rates[4,5]. A reason for this variation could lie in the variation in therapeutic services performed by the GPs themselves, such as minor surgery and cyriax injections. However, little is known about the effects of GP services on referral behaviour. In this paper we will investigate whether GPs that perform more therapeutic services, generate lower hospital care costs, i.e. lower referral rates.

Research that focuses on the effects of the numbers of GP services on referral behaviour is scarce and the results are inconsistent[6]. In Denmark, Krasnik et al. found a decrease in the number of referrals when there was an increase in the number of GP services (after the introduction of a payment for specific services)[7]. In the Netherlands, Groenewegen found cross-sectional associations between performed services and referrals, (more services were associated with fewer referrals). This evidence was in relation to therapeutic services, such as stitching an open wound or incising an abscess, but not for diagnostic services or removal of cysts[8]. In comparison, in the UK, Lowy et al. found no reduction in the number of referrals with an increase in minor surgery services after the introduction of a reimbursement system for minor surgery[9]. However, these studies date back to 1990 and they did not take into account clustering of data within practices or analysed effects on aggregated level and they did not distinguish between diagnoses. All these factors could affect the applicability of these effects in relation to the current situation.

The purpose of this paper is to examine whether GPs do refer fewer patients to hospital care when they perform more therapeutic services. The study will undertake this investigation in relation to separate diagnoses and will correct for the clustering of GP practices. It will focus on minor surgery for dermatological problems. These problems represent one of the most common reasons for GP consultations and referrals to specialist care[10,11]. The following questions will be answered: To what extent do GPs refer fewer patients to hospital care when they perform more minor surgery? How do these rates of referral vary between specific diagnoses? Which factors influence this association?

## Methods

Data were used from electronic medical records (EMRs) from GP practices that participated in the Netherlands Information Network of General Practice (LINH)[11]. The LINH database holds longitudinal data on morbidity, prescriptions and referrals. Diagnoses are coded using the ICPC-classification (International Classification of Primary Care)[12]. The network is a dynamic pool of practices, with yearly changes in their composition. The effect of minor surgery in general practice on referrals was analysed using 2006 and 2007 data. Medical ethical approval was not required for this research.

Episodes of care were defined as the unit of analysis. An episode of care includes 'all encounters for the management of a specific health problem'[13]. For example, if a patient consulted the GP for sebaceous cysts at visit 1 and the patient was treated via surgery at visit 2, both visits are included in the episode of care. Episodes were constructed with the aid of EPICON, an algorithm to group ICPC-coded contact records from EMRs in general practice into episodes of care[14,15]. The effect of minor surgery on referral was analysed for four different diagnoses. These diagnoses represent the top four most frequently observed diagnoses for minor surgery: laceration/cut, neoplasm skin benign/unspecified, nevus/mole and sebaceous cyst. The difference between the diagnosis neoplasm skin benign/unspecified and nevus/mole is not clear-cut. GPs can record a mole as nevus/mole and as neoplasm skin benign/unspecified, and therefore, the included complaints and GPs' decision making process were expected to be similar in both diagnoses. For this reason, these diagnoses were grouped into one category: benign neoplasm skin/nevus.

Data were used from 48 GP practices with complete data on the registration of care-episodes,[16] claimed services, referrals and number of GPs (whole time equivalents (WTE)) working in the practice in 2006 and 2007. These practices form a representative sample of Dutch general practices with regard to practice type (solo, duo, group or health centre), degree of urbanisation and location (province). From these practices, patients (whose age and gender were known), who were undergoing certain care-episodes, were identified; these care-episodes were laceration/cut (ICPC: S18), benign neoplasm skin/nevus (S79/S82) or sebaceous cyst (S93). After the inclusion criteria, a total of 14203 patients and 15923 care-episodes were included in the analyses.

### Measurements

For each care-episode, GPs had three options: (I) to do nothing, i.e. no referral or minor surgery, (II) to perform minor surgery and (III) to refer patients to a medical specialist.

#### Referrals

Each episode was typed as 'referred' or 'not referred', dependent on whether a new referral had been issued in any of the contacts within this episode of care. Only referral to dermatology, surgery and plastic surgery were included.

#### Minor surgery

Each episode was typed 'minor surgery' or 'not minor surgery' dependent on whether or not minor surgery had been claimed in any of the contacts with this episode of care.

#### Covariates influencing the association

##### Distance to hospital

For each patient, distance to the closest hospital by road was assessed on the basis of postal codes. For a patient, the distance to the closest hospital might influence the association between minor surgery and referral rate to specialist care, since GPs might be more reluctant to refer patients living further away from a hospital[4].

##### Primary care nurse

The presence of a primary care nurse might influence the time available to perform minor surgery. GPs in a practice with a primary care nurse could delegate more tasks and therefore, have more time for minor surgery. Also, specialised primary care nurses may sometimes perform or assist with minor surgery.

##### GPs' workload

GPs' workload might negatively affect the number of minor surgery interventions. GPs' workload was defined as the weighted number of short and long consultations (weight of 1 and 2) and short and long home visits (weight of 1.5 and 2.5) per WTE GP working in the practice divided by 1000. As most of the GPs in this study (and in the Netherlands as a whole) are self-employed, we used a self-report of WTE; A whole working week is set at 5 days each consisting of two parts (morning and afternoon). GPs were asked to report the number of day parts they work in the practice.

In addition to factors that might influence the association between minor surgery and referral rate to specialist care, patients' age and gender were also taken into account.

### Statistical analyses

To analyse the effect of minor surgery on referral behaviour in general practice, multilevel multinomial regression analyses were conducted comparing three groups: (I) no referral or minor surgery, (II) minor surgery and (III) referral to dermatology, surgery or plastic surgery. Minor surgery (II) and referral to medical specialist (III) were regarded as treatment groups and were compared to 'no referral or minor surgery' (I). In the multilevel analyses two levels were distinguished: care-episodes within GP practices. No separate level for patients was discerned because very few patients had more than one episode of care. For these diagnoses, 5.4% to 9.2% of the patients had more than one episode.

For each diagnosis group, multilevel multinomial regression analysis was performed in two steps. In step one, crude multilevel multinomial regression analyses were performed with no covariates taken into account. In step two, covariates were added to the model to correct for differences in the practice population (age and gender) and assess the effect of the addition of factors. On the GP-practice level the influence of the GP-practice on the use of the therapy group is measured using per therapy group variances and a covariance between the therapy groups. Based on these variances and covariance we can measure the correlation, which represents the association between minor surgery and referrals at the GP-practice level. A negative correlation indicates that GP-practices that perform more minor surgery refer fewer patients. It is important to notice that this correlation is corrected for the covariates in the model. The GP-practice effects for minor surgery and referral are estimated by the model as two normally distributed variables (logit scale) with a mean (intercept) and a variance (sum of the GP-practice variance and covariance associated with that variable). To illustrate how the change in minor surgery leads to a change in referrals, we further analysed the correlation derived from the multilevel multinomial regression analyses using the following formula (Y - Y_mean_)/SD_Y _= r * (X - X_mean_)/SD_X _. Y is referral and X is minor surgery value (on the logit scale), SD is the standard deviation calculated as the square root from the sum of the variance and covariance (at the GP-practice level), and r is the correlation. After transforming the values back to the probability scale we can see how much percentage of change in referral is associated with percentage change in minor surgery. It is crucial to notice that this relation on the probability scale is nonlineair. This means (assuming a negative correlation) that if the referrals would change from 5% to 7% the minor surgery could go down with say 1.5%, but if the referrals would change from 1% to 3% the minor surgery could go down 0.5%. In addition, intraclass correlations (ICC's) and a 95% range on GP practice level (intercept plus and minus 1.96 times the square root of the between practice variation and transformed back from a logit scale) were calculated for all outcome measures. The association between the covariates and the two therapy groups is expressed using odds ratios (OR) and 95% confidence intervals (CI). The models were estimated using multilevel multinomial regression analyses, for unordered categories, with PQL (penalised quasi-likelihood), first order and constrained level I variance (MLwiN 2.02).

## Results

Table 1 describes the patient and practice characteristics. Patients with care-episodes of laceration/cut, benign neoplasm skin/nevus or sebaceous cyst had a mean age of 39 years (SD:21.4). On average, patients were living 8187 (SD:6452) metres away from a hospital. Almost two thirds of the GP practices had a primary care nurse working in the practice. GP practices performed minor surgery in 8.9% (SD:14.6) of the care-episodes with laceration/cut. This was 27.4% (SD:14.4) for benign neoplasm skin/nevus and 26.4% (SD:13.8) for sebaceous cyst. The referral rate differed strongly between the diagnoses. For care-episodes with a laceration/cut, 1.0% of the patients were referred to hospital care, whereas this was 10.2% for benign neoplasm skin/nevus and 8.2% for sebaceous cyst.

**Table 1:** patient and practice characteristics

**Patient level (n = 14203)**	
Distance to hospital (kilometres)^1 ^	8.19 (SD:6.45)
Age (years)^1 ^	39.2 (SD: 21.4)
Gender^2^	
Male	6908 (48.6%)
Female	7295 (51.4%)
**Practice level (n = 48)**	
Primary care nurse^2^	
Yes	29 (60.4%)
No	19 (39.6%)
Workload GP (consultation units/WTE/1000)^1 ^	6.32 (SD: 1.41)

^1 ^Mean (SD); ^2 ^Number (%)

Table 2 shows the referral rate for episodes with and without minor surgery. In general, referral rates were lower in care-episodes in which minor surgery was performed. For laceration/cut, only 0.7% of the cases with minor surgery had a referral to a medical specialist. For benign neoplasm skin/nevus and sebaceous cysts, this was 2.4% and 2.2% respectively. Without minor surgery, referral rates were much higher, especially for benign neoplasm skin/nevus and sebaceous cyst with a referral rate of 13.3% and 10.6% respectively. These results suggest that minor surgery indeed substituted for referrals. However, these results might also reflect differences in severity. For severe complaints, patients will probably be directly referred to the medical specialist. And for minor complaints, it is likely that no referral or minor surgery will be performed. Therefore, these results could be biased by the type of laceration/cut, sebaceous cysts or benign neoplasm skin/nevus which patients present to GPs. To take this into account, we analysed the effects of minor surgery on referral rate on the level of the general practice. Since, laceration/cuts, sebaceous cysts and benign neoplasm skin/nevus are common complaints we expected the severity of the cases to be equally spread over the practices.

**Table 2:** Number of care-episodes with and without minor surgery with the percentage (standard deviation) of referrals

	Minor surgery in disease episode			
	
	No		Yes	
	**Number of care-episodes**	**Percentage of referrals**	**Number of care-episodes**	**Percentage of referrals**

Laceration/cut	4440	1.1	815	0.7
Benign neoplasm skin/nevus	5373	13.3	2177	2.4
Sebaceous cyst	2220	10.6	899	2.2

### Relationship between percentage of minor surgery and referrals

Table 3 shows the results of the multilevel multinomial regression analyses for each diagnosis group. Since our model divided the care-episodes in (I) no referral or minor surgery, (II) minor surgery or (III) referral, care-episodes with both minor surgery and referral (see Table 2) were excluded from the multilevel multinomial regression analyses.

**Table 3:** Multilevel multinomial regression analyses for minor surgery and referral in comparison to no treatment

	Laceration/cut				Benign neoplasm skin/nevus				Sebaceous cyst			
	
	Empty model		Adjusted model		Empty model		Adjusted model		Empty model		Adjusted model	
	
	Minor surgery	Referral	Minor surgery	Referral	Minor surgery	Referral	Minor surgery	Referral	Minor surgery	Referral	Minor surgery	Referral
	
	Intercept (SE)		Intercept (SE)		Intercept (SE)		Intercept (SE)		Intercept (SE)		Intercept (SE)	
	
	-2.18 (0.18) - 10.2%	-4.74 (0.22) - 0.9%	-2.05 (0.15) 11.4%	-4.82 (0.23) 0.8%	-0.94 (0.12) 28.1%	-2.01 (0.13) 11.8%	-0.80 (0.10) 31.0%	-1.82 (0.12) 13.9%	-0.87 (0.09) 29.5%	-2.26 (0.12) 9.4%	-0.89 (0.09) 29.1%	-2.26 (0.12) 9.4%
			**OR (95% CI)**				**OR (95% CI)**				**OR (95% CI)**	

Gender, female			0.85 (0.72-1.01)	1.23 (0.68-2.20)			0.94 (0.85-1.05)	1.13 (0.96-1.33)			**0.62 (0.53-0.73)**	**0.76 (0.58-0.99)**
Age			**1.01 (1.01-1.01)**	1.01 (1.00-1.02)			**1.01 (1.01-1.01)**	**0.99 (0.99-1.00)**			**1.01 (1.01-1.02)**	1.00 (0.99-1.01)
Distance to hospital			**1.00 (1.00-1.00)**	1.00 (1.00-1.00)			1.00 (1.00-1.00)	1.00 (1.00-1.00)			1.00 (1.00-1.00)	1.00 (1.00-1.00)
Primary care nurse			1.69 (0.96-2.99)	1.66 (0.64-4.28)			**1.49 (1.06-2.09)**	1.43 (0.93-2.18)			1.03 (0.71-1.49)	1.32 (0.80-2.18)
Workload			1.00 (1.00-1.00)	1.00 (1.00-1.00)			1.00 (1.00-1.00)	1.00 (1.00-1.00)			**1.00 (1.00-1.00)**	1.00 (1.00-1.00)

	Variance (SE)		Variance (SE)		Variance (SE)		Variance (SE)		Variance (SE)		Variance (SE)	

Between GP practice variance	1.25(0.30)	0.76 (0.41)	0.80 (0.21)	0.74 (0.40)	0.57 (0.13)	0.67 (0.16)	0.40 (0.09)	0.51 (0.13)	0.32 (0.09)	0.38 (0.14)	0.27 (0.08)	0.33 (0.13)
Covariance	-0.26 (0.27)		-0.29 (0.22)		0.11 (0.10)		-0.12 (0.08)		-0.11 (0.08)		-0.13 (0.07)	

	Correlations		Correlations		Correlations		Correlations		Correlations		Correlations	

Correlation GP practice variances 'therapy groups'^a^	-0.27 (-0.51 - 0.02)		**-0.38 (-0.60- -0.11)**		0.18 (-0.44--0.11)		-0.26 (-0.506 - 0.03)		**-0.31 (-0.55 - -0.03)**		**-0.42 (-0.63 - -0.16)**	

ICC	27.5	18.7	19.6	18.3	14.9	16.8	10.8	13.5	8.7	10.3	7.7	9.2

95% range on GP practice	1.6-44.3%	0.2-3.4%	3.1-34.4%	0.2-2.9%	7.2-66.3%	2.3-43.1%	13.7-55.9%	4.5-35.5%	14.6-49.3%	3.6-22.4%	16.5-46.1%	4.2-20.0%

*Based on multilevel multinomial regression analyses for minor surgery and referral rate in comparison to no treatment in care-episodes of laceration/cut, benign neoplasm skin/nevus and sebaceous cyst.

^a ^This is the correlation between the variances of the two defined therapy groups (minor surgery and referral) at GP level

^b ^because we used nominal data the variance at the lowest level (episodes) is not determined but given by π^2^/3

^c ^calculated by intercept plus and minus 1.96 times the square root of the between practice variation and transformed back from a logit scale).

Results show that minor surgery is more often performed in older patients for all diagnosis groups. There is no age effect on referrals, except for benign neoplasm skin/nevus (0.99;CI:0.99-1.00). For benign neoplasm skin/nevus, minor surgery is more often performed in older patients and fewer of them are referred. The presence of a primary care nurse only affects the number of minor surgery interventions for benign neoplasm skin/nevus (1.49;CI:1.06-2.09). Women have a smaller likelihood of minor surgery (0.62;CI0.53-0.73) and a smaller likelihood of referral (0.76; CI:0.58-0.99) for sebaceous cysts. So, it seems that for sebaceous cysts, males rather than females more often receive treatment in the form of minor surgery or referral.

Table 3 also shows the correlation between minor surgery intervention and referrals to a medical specialist at GP practice level. There is a significant negative correlation for laceration/cut (-0.38;CI:-0.599 - -0.108) and sebaceous cyst (-0.42;CI:-0.629- -0.16), but not for benign neoplasm skin/nevus (-0.26;CI:-0.506-0.03). This means that for laceration/cut and sebaceous cyst care-episodes, GP practices that perform more minor surgery refer fewer patients to a medical specialist. The correlations were affected by the addition of the covariates. To look into the effects of the separate covariates on the correlations between minor surgery intervention and referrals to a medical specialist at GP practice level for laceration/cut and sebaceous cyst, analyses were performed with and without the covariates included in the analyses. The presence of a primary care nurse and GPs' workload affected the correction negatively, i.e. the correlation showed a higher negative correlation when these variables were included in the analyses. In addition, distance to hospital affected the correlation for laceration/cut positively, i.e. addition of the factor showed a correlation closer to zero, and thereby explained part of the relation. Age and gender hardly affected the correlation between minor surgery and referral rate.

**Figure 1:**
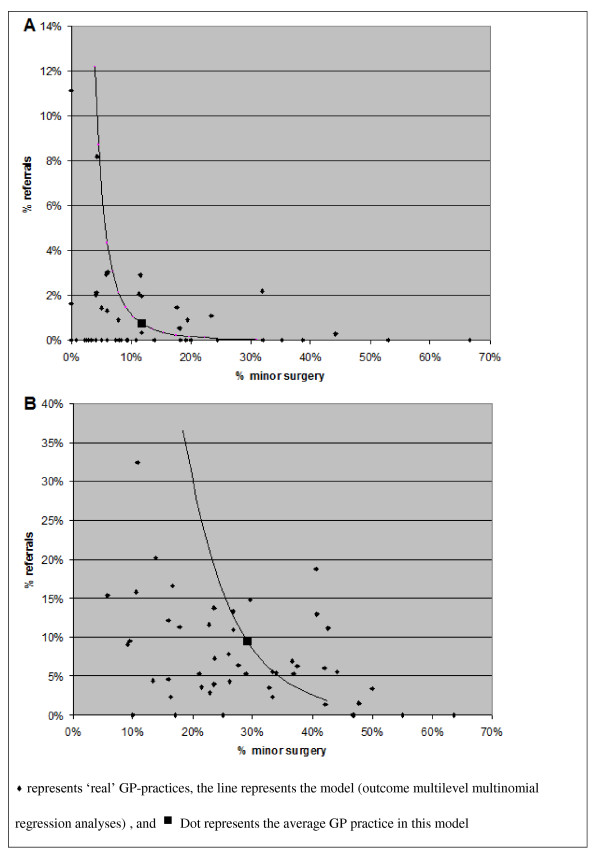
Relation between minor surgery interventions and referral rate on the level of GP-practice*. *Results based on the calculated correlations between minor surgery intervention and referral rate in general practice in multilevel multinomial regression analyses for care-episodes of laceration/cut (A) and sebaceous cyst (B).

To illustrate the clinical relevance of these differences, Figure 1 shows the absolute percentage of minor surgery interventions and referrals for care-episodes of laceration/cut and sebaceous cyst. The 'dot' in the figure represents the average GP practice. The correlations for laceration/cut and sebaceous cyst are similar; however, the size of the absolute effect differs. For the average GP practice, performing minor surgery in 5% more of the care-episodes of laceration/cut (from 11.4% to 16.4%), changes the referral rate from 0.8% to 0.3%. In comparison, in care-episodes of sebaceous cysts, 5% more minor surgery interventions (from 29.1% to 34.1%) changes the referral rate from 9.4% to 5.1%. These results are based on association, and therefore, conclusion about cause-effect relationships can not be made.

## Discussion

Our findings indicate that the effects of minor surgery performed in general practice on the rate of referral to hospital care varied by diagnosis. Minor surgery was associated with fewer referrals to hospital care for sebaceous cysts and laceration/cuts, but not for benign neoplasm skin/nevus. However, the absolute difference in referral rate appeared to be only relevant for sebaceous cysts. For laceration/cuts, referral rates were generally low, in absolute terms, with a mean of 1%, whereas for sebaceous cysts the mean referral rate was 8.2%. If an average general practice performed 5% more minor surgery interventions in cases of sebaceous cysts, this would lead to a lowering of the referral rate of 4.3%.

Previous research is inconsistent with respect to effects of services provided in general practice on referrals to medical specialists. Krasnik et al.[7], Groenewegen [8] and Fleming[17] found an effect of (specific) GP interventions on the chance of referrals, whereas Lowy et al.[9] found no reduction in the chance of referrals, with an increase in minor surgery interventions. Our study found an effect of minor surgery on the number of referrals in two out of three diagnosis groups, and showed that effects on referrals were diagnosis specific. This variation in outcome between these studies may be caused by methodological differences. For example, Lowy et al. did not distinguish between diagnoses. Krasnik et al. and Fleming analysed the total contact and referral rates, without distinguishing between specific services or diagnoses. Groenewegen distinguished different services and diagnoses but this study was based on a limited number of care-episodes only.

The only relevant association between the number of minor surgery interventions performed in a practice and the chance of referral was found for sebaceous cysts. Performing more minor surgery for laceration/cuts and benign neoplasm skin/nevus in the GP practice did not have a (large) effect on the change of referral. What are the reasons for this difference between diagnoses?

For laceration/cuts, the magnitude of the correlation was in the same order as care-episodes of sebaceous cysts. However, the absolute change in referral rate was small. This was due to the low overall referral rate for laceration/cuts. Mostly, GPs will see non-urgent problems, because usually, patients with serious lacerations/cuts will go directly to hospital emergency departments. Therefore, the overall referral rate for laceration/cuts is low, namely 1.0%.

On the other hand, referral rates for benign neoplasm skin/nevus are high, with an average of 10.2% and minor surgery is often performed for this diagnosis. So, in care-episodes of benign neoplasm skin/nevus, enough room exists for improvement, but still no effect was found. The reason for this could be that the treatment for sebaceous cysts is more straightforward than for benign neoplasm skin/nevus. This is supported by the smaller variation in referral rates between practices (see Table 3). There is less professional uncertainty in the treatment of sebaceous cysts than for neoplasm skin/nevus[18]. Sebaceous cysts hardly ever become malign, whereas research has reported that approximately 25% of melanomas are historically associated with a pre-existing nevus[19]. In addition, research has shown that GPs do not always recognise skin malignancies, or inadequately excise neoplasm of the skin[20-22]. More often than not, GPs will perform minor surgery for benign neoplasm without suspicion of malignancies. This is also the case where no referral is needed. So minor surgery is probably mostly performed for cases of benign neoplasm skin/nevus where no room for improvement in referral rate exists. In the case of sebaceous cysts, risks are lower and therefore, room for improvement in terms of referral rate to medical specialist is present.

For sebaceous cysts, males rather than females more often receive treatment in the form of minor surgery or referral. An explanation could lie in the GP visiting behaviour of women with sebaceous cysts. Woman could visit the GP more frequently for aesthetic reasons, when treatment is not necessary. However, incidence rate did not differ between men and women, and therefore, does not confirm this explanation.

### Policy relevance

Theoretically, performing five more minor surgery interventions per 100 care-episodes would result in 4.3 fewer referrals for sebaceous cysts. In the Netherlands, the fee for minor surgery ranges from €51 to €76.5 in general practice and €136.50 to €458.05 in hospital settings. So, five more minor surgery interventions would cost €255 - €382.5 and save €587-€1969.6. In the UK, National Health Service (NHS) reference costs of minor surgery in general practice is £449.74 (SD:47.74) and £1222.24 (SD:23.24) for minor surgery in hospital care settings[23]. In the UK, an increase of 5% in GP minor surgery interventions for sebaceous cysts would result in a saving of about £3000. These calculations are based on a business-cost perspective and do not include potential consequences from diagnostic error from a societal perspective nor does it include indirect costs. However, it should be noted that performing minor surgery requires specific skills, which will not be present in all GP surgeries. So, stimulating GPs to perform more minor surgery may lead to an increase in GP practices that already perform more minor surgery interventions, and where the monetary gain is much less than in the average GP practice. Further, it should be mentioned that treating patients in general practice has additional advantages, over hospital settings, in terms of travel time and continuity of care. Another option to save resources could be to organize joint courses on the workplace, thereby improving alliance between GPs and hospital specialists and improving minor surgery in primary care. A review of Akbari et al. showed that active local educational interventions involving secondary care specialists can impact on referral rates [24].

### Strengths and limitations of the study

This study was based on a large dataset representing GP data relating to consultations, morbidity and referrals based on EMRs. This enabled us to analyse the effect of minor surgery on referrals for specific diagnosis groups and correct for the clustering of referrals within practices using multilevel analysis. However, our study has some limitations. First, only data were available on minor surgery for which money was claimed and not on minor surgery actually performed. GPs might perform more minor surgeries for which no money is claimed. This may have affected the associations in both directions. In addition, the severity of the episodes was unknown and may have influenced the association between minor surgery and referrals. We tried to solve this by determining the correlation between minor surgery and referrals on the level of the GP practice, with the assumption that the severity of care-episode did not differ between the practices. On average 65 (sebaceous cyst) to 157 (benign neoplasm skin/nevus) care-episodes occurred per practice per year, which, it is suggested, should be enough to level out differences in severity.

## Conclusions

Our study shows that the effect of minor surgery on the chance of referral was diagnosis specific. Patients with sebaceous cysts had a lower chance of referral if GP practices perform more minor surgery. No (great) effects of minor surgery were found for benign neoplasm skin/nevus and lacerations/cut. Encouraging GPs to perform more minor surgery interventions for patients with sebaceous cysts has the potential to prevent specialist referrals and cost reduction. Future research is required to explore the cost-effectiveness of minor surgery in detail.

## Competing interests

The authors declare that they have no competing interests.

## Authors' contributions

CD, RV, PG and DB were involved in the conception of the research question

CD and PS were involved in analysing the data. All authors had full access to all the data and contributed to the interpretation of the data. CD drafted the manuscript, which was reviewed by all authors.

## Pre-publication history

The pre-publication history for this paper can be accessed here:

http://www.biomedcentral.com/1472-6963/11/2/prepub
